# Nutritional, antioxidant, and sensory properties of *Ziziphus spina-christi* fruit powder and its application in bakery products

**DOI:** 10.1038/s41598-025-30233-9

**Published:** 2025-12-11

**Authors:** Naglaa A. A. Hassan, Arig W. Elkhouly, Maha I. K. Ali

**Affiliations:** 1 Department of Home Economics, Faculty of Agriculture, Alexandria University, Alexandria, Egypt; 2 Food Industry Technology Program, Faculty of Industrial and Energy Technology, Borg Al Arab Technological University (BATU), Alexandria, Egypt; 3 Department of Special Food and Nutrition, Food Technology Research Institute, Agricultural Research Center, Giza, Egypt

**Keywords:** Ziziphus spina-christi, Nutritional composition, DPPH radical scavenging, Texture analysis, Functional food, Biochemistry, Health care, Plant sciences

## Abstract

Bioactive compounds from natural sources have attracted growing scientific interest due to their functional, pharmacological, and biological properties. Among these, sidr (Ziziphus spina-christi), a plant native to South and East Asia, is recognized for its nutritional and therapeutic potential. Z. spina-christi produces fruits rich in bioactive constituents, yet they remain largely underutilized in food applications. This study investigated the effect of fortifying waffles and breadsticks with dehydrated Z. spina-christi fruit powder (DNP) on their nutritional composition, antioxidant activity, and sensory characteristics. Wheat flour was partially replaced with DNP at 0%, 5%, 10%, 15%, and 20% (w/w). Adding DNP significantly (*P* < 0.05) increased ash, crude fiber, total phenolic and flavonoid contents, and DPPH radical scavenging activity, while slightly decreasing protein content and lightness (L*) values. The redness (a*) increased due to the fruit’s natural pigments, and texture parameters like hardness, gumminess, and chewiness rose with higher substitution levels. Sensory evaluation revealed that 10–15% DNP provided the optimal balance between improved nutritional and antioxidant properties and acceptable sensory quality, whereas higher levels slightly reduced overall acceptability. Therefore, this study recommends the use of Ziziphus spina-christi fruit powder as a functional ingredient in bakery products, enhancing their health-promoting properties without compromising consumer acceptance.

## Introduction

In recent years, consumer preferences have increasingly shifted toward healthier and more natural foods, driven by greater awareness that improved dietary habits can enhance overall health and well-being^1,2^. This trend has encouraged the food industry to develop innovative products that not only supply essential nutrients but also provide additional health benefits. Consequently, functional foods have emerged, offering bioactive compounds, probiotics, unsaturated fatty acids, and dietary fiber, all of which contribute to improved health and a lower risk of chronic diseases^3^.

Currently, global research and development strategies have increasingly focused on the use of edible wild fruits in functional foods, particularly those traditionally recognized in folk medicine by rural communities. The genus Ziziphus (Family: Rhamnaceae) includes approximately 53 species native to Africa, Australia, and Asia. Among them, Ziziphus spina-christi L. (Christ’s thorn jujube) is an evergreen tree native to Sudan and widely distributed along the Nile in Khartoum and the Nile Valley of Egypt, where it is commonly known as sidr or nabaq^4^.

Sidr (Ziziphus spina-christi), commonly known as nabaq, is traditionally consumed in fresh, dried, and cooked forms^5^. Historical records highlight its significance in ancient Egypt, where it was considered a valuable component of the native flora with applications in medicine, nutrition, and ritual practices. The plant, referred to as ‘Nebes’ in ancient Egyptian texts, was cited in 33 medicinal prescriptions documented in medical papyri, underscoring its long-standing ethnopharmacological importance. Moreover, Nebes and a type of bread generated from it were among important food offerings given to the deceased inside tombs along with the main staple cereals, fruits, and beverages of the ancient Egyptians. This tree did not merely qualify for being an important feature of the indigenous wild flora of Egypt but it was also grown in the ancient Egyptian gardens^6^. Moreover, it has been widely recognized for its multifunctional properties and valued role in traditional medicine^7^. This species is now quite widely spread throughout Mediterranean area in Africa, China, India, Australia and Tropical America^8^.

Ziziphus fruits are rich in bioactive compounds such as alkaloids, flavonoids, saponins, tannins, terpenoids, and glycosides, which contribute to their diverse pharmacological effects, including antioxidant, antimicrobial, antifungal, antihypertensive, antidiabetic, and anticancer activities, along with hypoglycemic, anti-inflammatory, hepatoprotective, and immune-enhancing properties^9,10^.

The present study provides a novel and systematic evaluation of Ziziphus spina-christi L. (Sidr or Nabaq) fruit powder as a functional ingredient in bakery products an area that has received very limited scientific attention. In Egypt, this valuable fruit remains largely underutilized and is not yet widely cultivated, despite its remarkable drought tolerance, adaptability to arid conditions, and high nutritional and antioxidant potential. These characteristics make it a promising crop for sustainable agriculture and functional food innovation. While most previous research has mainly addressed the medicinal or basic nutritional aspects of Sidr, the current study uniquely investigates the impact of partially substituting wheat flour with 5–20% dehydrated Sidr fruit powder on the nutritional composition, antioxidant activity, color, texture, and sensory characteristics of waffles and breadsticks. By integrating chemical, functional, and sensory evaluations, this work demonstrates the potential of Z. spina-christi fruit powder to enhance the nutritional profile and sensory quality of bakery products, while also supporting its future cultivation and industrial utilization in Egypt.

## Materials and methods

### Materials

Fresh ripe fruits of *Ziziphus spina-christi* (locally known as nabaq or sidr) were purchased from the local market in Alexandria, Egypt, during the harvesting season of 2024. Along with nabaq, other ingredients including wheat flour (72% extraction corresponding to refined wheat flour with reduced bran content, commonly used for bakery products), sugar, eggs, dry yeast, spice mixture (onion powder, cumin, white pepper), milk, vanilla, vinegar, corn oil, salt, and packing materials were also obtained from local markets in Alexandria, Egypt. All chemicals and reagents used in the study were of analytical grade and supplied by Sigma Company (USA).

### Dehydrated nabaq powder preparation

Fresh nabaq fruits (10 kg) were thoroughly washed with potable tap water. The pulp was manually separated from the seeds using a stainless-steel knife. The pulp was dehydrated in a thermostatically controlled hot air oven (UN750, Memmert, Germany) at 45 °C for 12 h. The dried pulp was then ground into a fine powder using a spice mill (AR1044, Moulinex, France; 180 W) and sieved using a 40-mesh screen, after that, stored in air-tight glass jars at 4 °C until analysis.

### Preparation of waffle

Waffles were prepared following the method of Kigozi, et al.^11^. with partial substitution of wheat flour by nabaq powder at 5, 10, 15, and 20% (w/w) (Table 1). All ingredients were mixed using a Kenwood Major Titanium mixer (Kenwood, Japan). The batter was allowed to stand for a few minutes, then baked in a preheated waffle maker at 150 °C for 3.5 min. Baked waffles were immediately packed in sealed bags to maintain crispness.

### Breadsticks preparation

Breadsticks were prepared according to El-Hadidy, et al.^12^, with wheat flour partially substituted by nabaq powder at 5, 10, 15, and 20% (w/w). Sugar and instant dry yeast were dissolved in warm water, while flour was mixed with salt and oil. Dough was kneaded manually until soft and smooth, then placed in a plastic bag and fermented at 30 ± 2 °C for 30 min. The dough was cut into sticks, proofed again for 30 min, and baked at 150 °C for 45 min on ungreased trays.

**Table 1:** Components utilized in the formulation of waffles and breadsticks.

Components	Products Formulations				
Waffle	W0 (Control)	WN1 (5% DNP)	WN2 (10% DNP)	WN3 (15% DNP)	WN4 (20%DNP)
Wheat flour	31.75	30.16	28.58	26.99	25.40
DNP	0.00	1.59	3.17	4.76	6.35
Egg	15.87	15.87	15.87	15.87	15.87
Sugar	22.22	22.22	22.22	22.22	22.22
Salt	0.64	0.64	0.64	0.64	0.64
Liquid milk	28.57	28.57	28.57	28.57	28.57
Vanilla	0.95	0.95	0.95	0.95	0.95

Breadsticks	BS0 (Control)	BSN1 (5% DNP)	BSN2 (10% DNP)	BSN3 (15% DNP)	BSN4 (20% DNP)
Wheat flour	83.60	79.42	75.24	71.06	66.88
DNP	0.00	4.18	8.36	12.54	16.72
Salt	0.40	0.40	0.40	0.40	0.40
Yeast	0.50	0.50	0.50	0.50	0.50
Sugar	6.70	6.70	6.70	6.70	6.70
Corn oil	8.40	8.40	8.40	8.40	8.40
Spice	0.40	0.40	0.40	0.40	0.40

DNP (Dehydrated Nabaq powder).

### Chemical composition

The proximate composition of waffle and breadstick samples was determined according to the AOAC^13^methods. Moisture, ash, crude fiber, protein, and fat contents were analyzed, while total carbohydrate content was calculated by difference on a dry-weight basis using the formula: Total carbohydrates (%) = 100 − (protein + fat + ash + crude fiber). The total energy value (kcal) was calculated using the AOAC formula:

$$\:Energy\:content\:\left(K.cal\right)=4\left[\text{p}\text{r}\text{o}\text{t}\text{e}\text{i}\text{n}\:\left(\text{g}\right)+carbohydrates\:\left(g\right)\right]+\left[9*fat\left(g\right)\right]$$

### Preparation of extracts for antioxidant analysis

Sample extracts were prepared following the method of Öztürk, et al.^14^. Briefly, 5 g of powdered sample was homogenized in 25 mL of 75% methanol using a digital homogenizer (PRO25D, Thomas Scientific, USA). The homogenate was centrifuged at 7000 rpm for 15 min at 4 °C (Sigma 113, VWR International, Darmstadt, Germany). The supernatants were filtered through Whatman No. 1 filter paper and stored at 4 °C until analysis.

### Total phenolic content (TPC)

TPC was determined according to Abirami, et al.^15^. using the Folin–Ciocalteu method. Briefly, 0.2 mL of methanol extract was mixed with diluted Folin–Ciocalteu reagent (1:10) and incubated for 4 min. Then, 0.8 mL of 7.5% sodium carbonate solution was added, and the mixture was incubated at room temperature for 30 min. Absorbance was measured at 765 nm using a UV–Vis spectrophotometer (PG Instruments, Model T80+, Leicestershire, UK). A calibration curve was prepared using gallic acid as a standard, and the results were expressed as mg gallic acid equivalents (GAE) per g of dry weight (DW).

### Total flavonoids

Total flavonoid content was estimated following the method of Barros, et al.^16^. A 0.5 mL extract was mixed with 2 mL distilled water and 150 µL of 5% sodium nitrite. After 6 min, 150 µL of 10% aluminum chloride was added, followed 6 min later by 2 mL of 4% NaOH. The mixture was diluted to 5 mL with distilled water and incubated for 15 min. Absorbance was recorded at 510 nm using a UV–Vis spectrophotometer. A calibration curve was constructed using rutin as a standard, and the results were expressed as mg rutin equivalents (RE) per g of dry weight (DW).

### Antioxidant activity (DPPH assay)

The antioxidant capacity of extracts was assessed using the DPPH radical scavenging assay as described by Brand-Williams, et al.^17^. Extracts were mixed with 2 mL of DPPH solution and kept in the dark at room temperature for 30 min. Absorbance was measured at 517 nm. The radical scavenging activity was expressed as percentage inhibition using the following equation:

$$\:Scavenging\:activity\:\left(\%\right)=\left(\text{As}\:\text{control}\:-\:\text{Abs}\:\text{sample}\:/\:\text{Abs}\:\text{control}\right)\times\:100$$

### Dietary fiber fractions

Neutral Detergent Fiber (NDF), Acid Detergent Fiber (ADF), and Acid Detergent Lignin (ADL) were determined according to AOAC^13^. Cellulose and hemicellulose were calculated as:

- Cellulose (%) = ADF (%) – ADL (%)
- Hemicellulose (%) = NDF (%) – ADF (%)

### Minerals contents

Minerals including P, Mg, Fe, K, Ca, Na, Cu, and Zn were determined according to AOAC^13^. Approximately 0.5 g of dehydrated Nabaq powder was accurately weighed into Teflon digestion tubes, and 5 mL of concentrated nitric acid (65% w/w, 14 mol L⁻¹) was added. The mixture was vortexed for 30 s and then digested in a microwave digestion system (Peeked, Shanghai, China) under the following conditions: 120 °C for 5 min, 150 °C for 10 min, and 190 °C for 20 min. After cooling to room temperature, the digests were diluted to 50 mL with deionized water. Mineral concentrations were quantified using inductively coupled plasma–optical emission spectrometry (ICP-OES, Agilent 5100 VDV, USA).

### pH measurement

The pH of samples was determined following Goulas and Kontominas^18^. using a digital pH meter (Mettler Toledo MP230, Switzerland).

### Sugar analysis

Total sugars were determined using the phenol–sulfuric acid method Ranganna^19^. Reducing sugars, glucose, and starch were determined by AOAC^13^ methods. Non-reducing sugars were calculated by subtracting reducing sugars from total sugars, and sucrose was estimated by multiplying non-reducing sugars by 0.95. Fructose was determined following the method of Jacobs^20^.

### Ascorbic acid content

Ascorbic acid content was determined using the 2,6-dichlorophenol indophenol (DCPIP) titrimetric method as described by AOAC^13^. Briefly, 10 g of dehydrated Nabaq powder was homogenized with 50 mL of 3% metaphosphoric acid solution and filtered through Whatman No. 1 filter paper. A 10 mL aliquot of the filtrate was titrated against the standardized DCPIP dye solution until a persistent light pink color appeared that lasted for at least 15 s, indicating the endpoint. A standard ascorbic acid solution was used to prepare the calibration curve, and the ascorbic acid content of the samples was calculated and expressed as mg/100 g dry weight (DW).

### Color measurement

The attributes of color include lightness (L*), redness (a*), and yellowness (b*) as measured using a Hunter Lab (Ultra Scan(VIS model colorimeter, USA)., according to Santipanichwong and Suphantharika^21^.Each parameter was recorded as the mean of five separate readings. The overall color difference between samples was calculated using:

$$\:E=\sqrt{{\left(a1-a2\right)}^{2}+\:{\left(b1-b2\right)}^{2}{+\:\left(L1-L2\right)}^{2}}$$

### Texture profile analysis

The texture profile values of the samples were measured using a TA-XT2 Texture Analyzer (Texture Pro CT3 V1.2, Brookfield, Middleboro, USA), following Yuan and Chang^22^. A load cell of 5 kg was used to produce force-time deformation curves at 1 mm/s cross-head speed. The texture parameters evaluated were hardness, chewiness, cohesiveness, springiness, gumminess, and resilience.

### Sensory evaluation

The Sensory evaluation was conducted with thirty panelists from the Department of Food Science and Technology, Faculty of Agriculture, Alexandria University. The panelists evaluated waffles and breadsticks for odor, color, flavor, and overall acceptability using a 9-point hedonic scale, where 1 = “extremely disliked” and 9 = “extremely liked”^23^.All samples were served simultaneously on white disposable plastic plates, with the order of presentation randomized across panelists. To minimize carry-over effects, water was provided for mouth rinsing between samples.

### Statistical analysis

Data were analyzed using the General Linear Model (GLM) procedure in **SAS software**,** version 9.1 (SAS Institute Inc.**,** Cary**,** NC**,** USA)**^24^. One-way ANOVA was applied, and Duncan’s multiple range test was used to compare means at *p* ≤ 0.05.

## Results and discussion

### Chemical composition of dehydrated nabaq powder

Table 2. presents the chemical composition of dehydrated Nabaq powder, including moisture (16.23%), protein (5.86%), fat (1.06%), ash (5.54%), fiber (4.21%), total carbohydrates (83.33%), and energy value (366.58 Kcal/100 g). The carbohydrate content of dehydrated nabaq powder (83.32%) was consistent with the findings of Abdelmuti^25^,Li, et al^26^. but lower than the 90.94% reported by Hashem and Abd El-Lahot^27^ found that moisture, protein, fat, ash, fiber, total carbohydrates, and total calories in dehydrated nabaq fruit were 17.56%, 4.58%, 0.59%, 3.89%, 2.51%, 90.94%, and 383.18 Kcal/100 g, respectively. Additionally, the ash content observed in the current study was lower than the 7.92% reported by Adekunle and Adenike^28^, who also found a fiber content of 6.09% in Nabaq fruit. Variations in proximate composition could be attributed to variety, agro-climatic conditions, ripening stage, harvesting time, and environmental influences^29^. Hashem and Abd El-Lahot^27^ found nabaq fruit to be an excellent energy source, with a calorific value of 387.39 Kcal per 100 g. Moreover, Altamim^30^ reported the chemical composition of Saudi Sidr (SS) and Egyptian Sidr (ES). The analysis showed that ES powder contained higher levels of ash (4.32%), protein (5.11%), fiber (11.58%), and lipids (1.05%) compared to SS powder, which, in contrast, had a greater proportion of total carbohydrates (83.63%. 77.94%).

Table 2. presents the bioactive properties of dehydrated Nabaq powder, which contains 25.27 mg GAE/g of total phenolic content, 214.68 mg RE/g of total flavonoid content, and a DPPH inhibition activity of 45.94%. The antioxidant activity observed in this study was lower than the values reported by Tawfek, et al.^31^. whereas the total flavonoid content was comparatively higher. The variation in total phenolic and flavonoid contents across studies may be attributed to several factors, including differences in fruit variety, geographical origin, soil and climatic conditions, maturity stage at harvest, and post-harvest handling. In addition, the type of solvent and extraction method used can greatly influence the recovery of phenolic and flavonoid compounds. Such factors may explain why our study reported comparatively higher flavonoid content but lower antioxidant activity relative to some previous reports. According to Hashem and Abd El-Lahot^27^, the Nabaq pulp had the most flavonoids (180.01 mg CE/g extract) and had a DPPH inhibition of 41.42%. As primary antioxidants, phenolic and flavonoid compounds are involved in scavenging and neutralizing free radicals, thus preventing the development of diseases, including cancer^32^. Moreover, different parts of the Nabaq fruit, including its pulp and seeds, have potential applications as nutraceutical ingredients in functional and health-promoting food products^33^. Similarly, our findings align with those of Al-Jassabi and Abdullah^34^, where antioxidant activity had been found to vary from 31.76% to 90.23%. Most of the antioxidant properties of Zizyphus belong to a dihydroxylated β-ring catecholate, which effectively donates hydrogen electrons to stabilize radical species^35^. Altamim^30^ reported significant (*p* < 0.05) differences in the phytochemical composition of Saudi Sidr (SS) and Egyptian Sidr (ES) powders, particularly in their total phenolic content (TPC) and total flavonoid content (TFC). The TPC of SS was higher than that of ES, suggesting that Saudi fruits may possess greater antioxidant potential due to their elevated phenolic levels. Phenolic compounds are well known for their antibacterial, anti-inflammatory, and antioxidant activities, which contribute to improved health and well-being. Similarly, flavonoids, a key subclass of phenolic compounds, are recognized for their potent antioxidant properties and health-promoting effects, including anticancer and cardioprotective benefits. The higher flavonoid content in SS highlights its potential to provide superior health benefits compared to ES, making it a more promising candidate for functional food applications.

The dietary fiber composition of dehydrated Nabaq powder (Table 2) confirms its richness in functional fibers. It contains 16.72% Neutral Detergent Fiber (NDF), 13.85% Acid Detergent Fiber (ADF), and 10.13% Acid Detergent Lignin (ADL), indicating a high proportion of structural polysaccharides. Cellulose (3.72%) and hemicellulose (2.87%) contribute to digestive health, while the relatively high lignin content enhances stool bulk and supports bowel function. Such natural fibers are valuable for maintaining gut health, strengthening immunity, and lowering the risk of chronic diseases including obesity, diabetes, cardiovascular disorders, and certain cancers^36^.

Findings demonstrated that dehydrated Nabaq powder contains significant amounts of essential minerals, including Fe (11.56 mg/100 g), Zn (6.31 mg/100 g), Ca (176.21 mg/100 g), Mg (66.73 mg/100 g), K (453.59 mg/100 g), Na (108.16 mg/100 g), Cu (1.14 mg/100 g), and Mn (2.48 mg/100 g), as presented in Table 2. These findings highlight the nutritional potential of Nabaq as a valuable dietary source of minerals, particularly calcium and potassium, which were present at remarkably high levels. The high potassium content is especially noteworthy, as this mineral plays a vital role in nerve transmission, muscle contraction, heartbeat regulation, nutrient transport into cells, and waste elimination. Moreover, diets rich in potassium can mitigate the adverse effects of sodium on blood pressure, underscoring the potential health benefits of incorporating Nabaq into daily nutrition^37^. Similarly, the significant presence of calcium, iron, and zinc further supports its value in maintaining bone health, oxygen transport, and immune function. When compared to earlier reports, our results indicate higher concentrations of Fe, Zn, Ca, and K than those reported by El Maaiden, et al.^33^, who found Ca, K, and Na contents ranging from 74.80 to 78.37 mg/100 g, 179.65–203.20 mg/100 g, and 22.77–33.70 mg/100 g, respectively. Tawfek, et al^31^. also described Nabaq as a nutritious fruit but reported substantially lower mineral values (Fe: 0.52 mg/100 g, Zn: 0.48 mg/100 g, P: 23.6 mg/100 g, K: 246 mg/100 g, and Ca: 25.4 mg/100 g). Similarly, USDA^38^ recognized Nabaq as a mineral-rich fruit, corroborating our findings. Hashem and Abd El-Lahot^27^further emphasized that dehydrated Nabaq pulp can supply significant proportions of daily dietary requirements, particularly Ca (15.42%), K (22.59%), Fe (63.75%), and Zn (106.5%), supporting its role as a natural supplement and blood health enhancer.

In contrast,, Altamim^30^ reported that Egyptian Sidr (ES) powder contained considerably higher levels of Fe and Zn (31.65 and 12.94 mg/100 g DM, respectively) than Saudi Sidr (SS) powder (21.66 and 9.03 mg/100 g DM, respectively). Such discrepancies between studies may arise from differences in geographical origin, soil fertility, climatic factors, genetic variability among cultivars, and post-harvest processing techniques, all of which can influence mineral accumulation in the fruit.

As reported in Table 2, the dehydrated Nabaq powder exhibited total acidity of 1.22% and a pH value of 4.13, with a high content of total sugars (70.19 g/100 g) specifically reducing sugars (41.48 g/100 g) and non-reducing sugars (28.71 g/100 g). The main sugars identified were sucrose (27.27 g/100 g), glucose (21.38 g/100 g), and fructose (15.72 g/100 g). The starch content was 19.24 g/100 g, and the fruit provided 15.77 mg/100 g of vitamin C. Compared with Abdelmuti^25^and Saied, et al.^4^, who reported lower values of total reducing sugars (22.6%), sucrose (21.8%), and glucose (9.6%), our findings revealed higher sugar concentrations, which may be attributed to differences in fruit maturity stage and environmental conditions influencing carbohydrate accumulation. Similarly, our results for acidity (1.22%) and pH (4.13) were very close to those reported by by Hashem and Abd El-Lahot^27^, who found a total acidity of 1.15% and a pH value of 3.95 in dehydrated Nabaq pulp. This similarity indicates consistency in the acidity profile of dehydrated Nabaq across studies. Regarding carbohydrate composition, Hashem and Abd El-Lahot (27) also reported high total sugars (71.08%), reducing sugars (43.52%), sucrose (27.19%), glucose (18.45%), starch content (18.26%), and vitamin C (18.95 mg/100 g), which are comparable to our findings. Minor variations in sugar and starch values between studies may be attributed to differences in fruit maturity, environmental conditions, and dehydration or analytical methods, which can influence carbohydrate retention and quantification.

**Table 2:** Proximate composition of dehydrated Nabaq powder. DNP (dehydrated Nabaq powder), *Total carbohydrate content was calculated on a dry-weight basis by difference, excluding moisture.

Properties	DNP	Properties	DNP
Moisture (%)	16.23 ± 0.09	Minerals content (mg/100 g)	
Crude protein (%)	5.86 ± 0.13	Fe	11.56 ± 0.48
fat (%)	1.06 ± 0.17	Zn	6.31 ± 0.34
Total ash (%)	5.54 ± 0.52	Ca	176.21 ± 1.23
Crude fiber (%)	4.21 ± 0.03	Mg	66.73 ± 0.67
Total carbohydrate^*^ (%)	83.33 ± 0.23	K	453.59 ± 1.64
Energy value (kcal/100 g)	366.58 ± 0.09	Na	108.16 ± 1.03
Bioactive compounds		Cu	1.14 ± 0.05
Total phenolic content (mg GA/g)	25.27 ± 0.45	Mn	2.48 ± 0.23
DPPH inhibition activity (%)	45.94 ± 0.48	Sugar Composition (g/100 g)	
Dietary fiber fractions%		Total sugars	70.19 ± 0.64
Acid detergent lignin (ADL)	10.13 ± 0.18	Reducing Sugars	41.48 ± 0.78
Acid detergent fiber (ADF)	13.85 ± 0.36	Non-Reducing Sugars	28.71 ± 0.45
Neutral detergent fiber (NDF)	16.72 ± 0.41	Sucrose	27.27 ± 0.34
Cellulose	3.72 ± 0.08	Glucose	21.38 ± 0.31
Hemicellulose	2.87 ± 0.13	Fructose	15.72 ± 0.39
Titritable Acidity (%)	1.22 ± 0.21	Starch	19.24 ± 0.28
pH value	4.13 ± 0.01	Vitamin C (mg/100 g)	15.77 ± 0.53

### Chemical composition of waffles, and breadsticks

Table 3 shows the chemical characteristics of the waffles. Incorporation of DNP significantly (*P* < 0.05) increased the ash, and crude fiber contents, which ranged from 1.26% to 2.69% and 2.05% to 2.13%, respectively. The highest values for ash, and crude fiber were observed in WN4, while the lowest were recorded in the control sample (W0). This enhancement may be explained by the naturally high fiber content of DNP, which contributed to elevated fiber levels in the supplemented treatments. The increase in ash and fiber is likely due to the abundance of minerals and dietary fiber in Nabq fruits, as previously reported by Hashem and Abd El-Lahot^27^, who emphasized the richness of Ziziphus spina-christi in Ca, K, and Fe, along with substantial fiber content. Comparable findings were reported by El Maaiden, et al.^33^, who demonstrated that Nabq fruit is a valuable source of dietary fiber capable of enhancing the nutritional quality of baked products.

No significant differences were observed in moisture content, crude ether extract, total carbohydrates, or energy value among the treatments. However, crude protein content generally declined with increasing levels of DNP, except in the WN2 and WN3 samples, with the lowest value (10.17%) recorded in WN4 as showen in Table 3. This reduction compared to the control (W0) may be explained by the dilution effect, as part of the protein-rich wheat flour was replaced with Nabq pulp, which contains relatively low protein. Similar findings were reported by Shukla, et al.^39^, who observed that muffins enriched with fruit- and vegetable-based ingredients rich in sugars and fiber but low in protein did not exhibit increased protein content, largely due to this dilution effect. In agreement with these results, the formulations in the present study also did not show an enhancement in protein levels. Additionally, a slight reduction in total fat content was observed across the treatments. In contrast, Altamim^30^ reported that incorporating Sidr pulp into protein bars significantly (*p* < 0.05) increased fiber (from 1.91% in the control to 4.72% at 15% ES). Abdelmuti^25^, who noted that dried Nabq is rich in sugars and starches, contributing to its high carbohydrate levels.

According to the results presented in Table 3, the incorporation of DNP significantly (*P* < 0.05) increased the moisture, ash, and crude fiber contents of the breadsticks, with ranges of 29.19–29.99%, 2.96–3.95%, and 1.53–2.16%, respectively. In contrast, protein content declined progressively as DNP levels increased, reaching the lowest value in BSN4 (20.62%). These outcomes are consistent with the findings of Hashem and Abd El-Lahot^27^, who stated that Nabaq pulp is particularly rich in ash and dietary fiber, thereby enhancing the nutritional quality of functional food products. Similarly, El Maaiden, et al^33^. confirmed the high mineral and fiber contents of Nabaq, which contribute positively to fortified foods. On the other hand, crude ether extract, total carbohydrates, and energy values were not significantly affected by DNP addition in any treatment. These findings align with Shukla, et al.^39^, who observed higher fiber content in baked goods enriched with fruit- and vegetable-based ingredients due to their naturally high fiber levels. Likewise, Altamim^30^, reported that replacing wheat flour with ES and SS powders increased moisture, ash, and crude fiber contents while showing negligible effects on protein and fat contents, supporting the present findings. The observed reduction in protein in this study may be attributed to the dilution effect, as protein-rich wheat flour was partially replaced by Nabaq pulp, which is lower in protein but richer in dietary fiber and minerals.

In general, the incorporation of *Ziziphus spina-christi* fruit powder led to a clear enhancement in the nutritional profile of the fortified bakery products. The fiber content significantly increased, which can contribute to improved digestive health and prolonged satiety. Furthermore, the elevated levels of vitamin C in the fortified samples highlight the contribution of the fruit’s natural antioxidant compounds. The addition of Sidr fruit powder also enriched the mineral composition, particularly calcium, zinc, magnesium, and potassium minerals essential for various metabolic and physiological processes. The higher vitamin C content may further enhance the bioavailability of these minerals, especially iron, thereby improving the overall nutritional efficacy and functional value of the fortified bakery products.

**Table 3:** Chemical composition of waffles and breadsticks. DNP(dehydrated Nabaq powder), W0 (Control), WN1 (5% DNP), WN2 (10% DMP), WN3 (15% DNP), WN4 (20%DNP), BS0 (Control), BSN1 (5% DNP), BSN2 (10% DNP), BSN3 (15% DNP), BSN4 (20% DNP). Values represent the means ± SD (on dry weight basis) of three independent replicates. Means within the same Colum followed by different letters are significantly different (*p* < 0.05).

Product	Moisture (%)	Crude protein (%)	fat (%)	Total ash (%)	Total carbohydrate (%)	Crude fiber (%)	Energy value (kcal/100 g)
Waffles							
W0 WN1 WN2 WN3 WN4	48.52 ± 0.41 ^a^ 48.75 ± 0.23 ^b^ 48.92 ± 0.21^ab^ 49.12 ± 0.40^ab^ 49.42 ± 0.24 ^a^	11.38 ± 0.11 ^a^ 11.06 ± 0.22 ^b^ 10.72 ± 0.05 ^c^ 10.46 ± 0.14 ^d^ 10.17 ± 0.06 ^e^	12.70 ± 0.04 ^a^ 12.69 ± 0.17 ^a^ 12.60 ± 0.12 ^a^ 12.56 ± 0.07 ^a^ 12.58 ± 0.17 ^a^	1.26 ± 0.04^e^ 1.51 ± 0.03^d^ 1.73 ± 0.06^c^ 2.02 ± 0.06^b^ 2.13 ± 0.05^a^	72.61 ± 0.08^a^ 72.50 ± 0.49^a^ 72.56 ± 0.23^a^ 72.49 ± 0.07^a^ 72.43 ± 0.22^a^	2.05 ± 0.06 ^d^ 2.24 ± 0.08 ^c^ 2.39 ± 0.08^bc^ 2.47 ± 0.06 ^b^ 2.69 ± 0.15 ^a^	450.26 ± 0.25^a^ 448.45 ± 0.49^a^ 446.52 ± 0.47^a^ 444.84 ± 0.35^a^ 443.62 ± 0.81^a^
Breadsticks							
BS0 BSN1 BSN2 BSN3 BSN4	29.19 ± 0.10 ^d^ 29.51 ± 0.08^cd^ 29.62 ± 0.11^bc^ 29.94 ± 0.34^ab^ 29.99 ± 0.20 ^a^	21.76 ± 0.08 ^a^ 21.57^a^ ± 0.09 21.17 ± 0.05^ab^ 20.94 ± 0.38 ^b^ 20.62 ± 0.44 ^b^	5.92 ± 0. 04 ^a^ 5.91 ± 0.05 ^a^ 5.88 ± 0.14 ^a^ 5.90 ± 0.03 ^a^ 5.87 ± 0.15 ^a^	2.96 ± 0.10^e^ 3.12 ± 0.05^d^ 3.44 ± 0.08^c^ 3.64 ± 0.07^b^ 3.95 ± 0.04^a^	67.83 ± 0.50 ^a^ 67.63 ± 0.15 ^a^ 67.66 ± 0.19 ^a^ 67.61 ± 0.41 ^a^ 67.40 ± 0.22 ^a^	1.53 ± 0.03 ^d^ 1.77^c^ ± 0.06 1.85 ± 0.04^bc^ 1.91 ± 0.02 ^b^ 2.16 ± 0.09 ^a^	411.64 ± 0.20^a^ 409.99 ± 0.58^a^ 408.24 ± 0.51^a^ 407.30 ± 0.31^a^ 404.91 ± 0.40^a^

### Bioactive compounds and antioxidant activity of waffles, and breadsticks

**Fig. 1:**
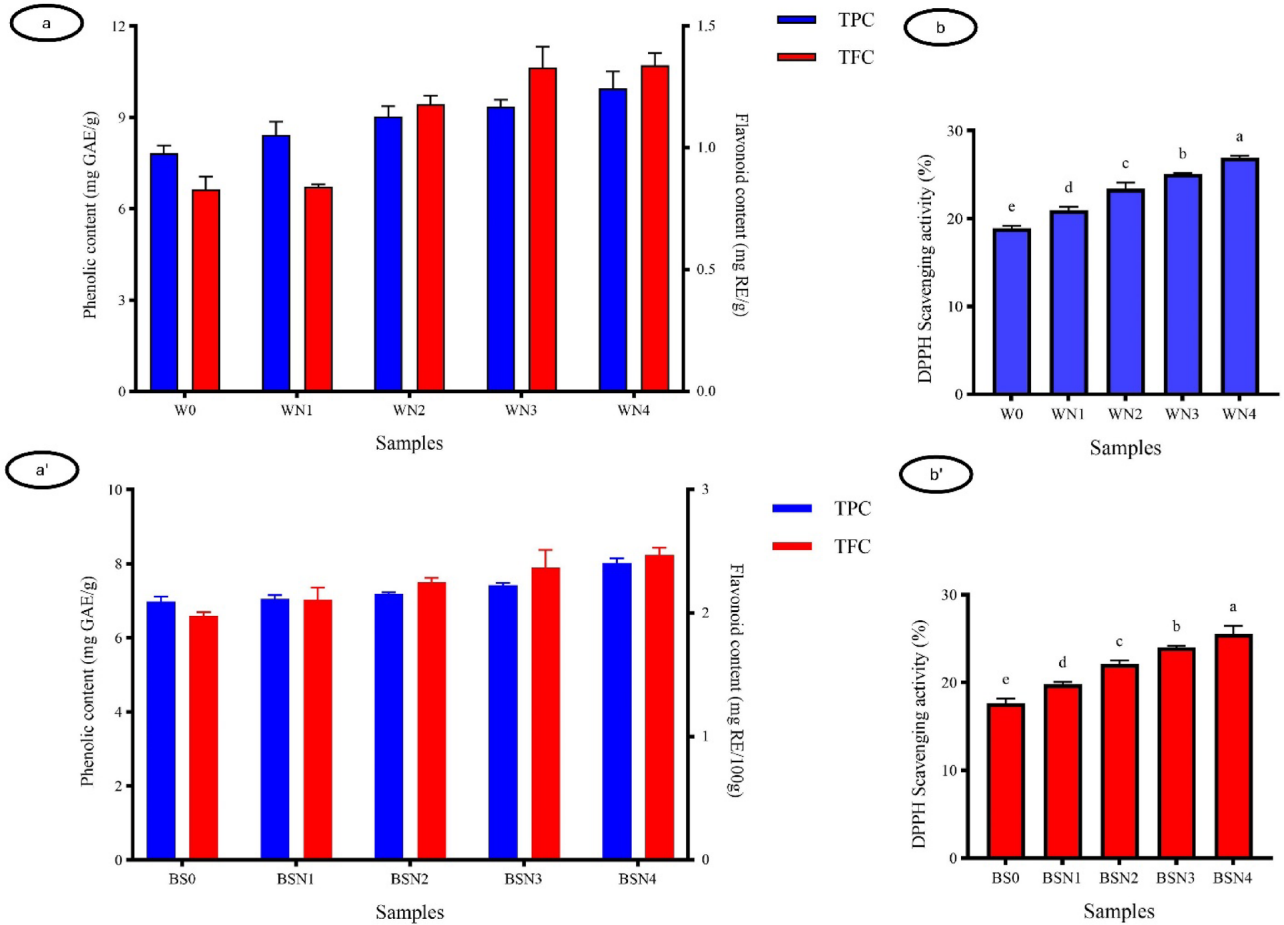
Total phenolic **(**TPC), Total flavonoid (TFC) contents and DPPH values of waffles **(a-b)**, Values represent the means ± standard deviation (*n* = 3). DNP = Dehydrated Nabaq powder, W0 (Control), WN1 (5% DNP), WN2 (10% DMP), WN3 (15% DNP), WN4 (20%DNP). TPC, TFC and DPPH values of breadsticks **(a**^**`**^**- b**^**`**^**)**, BS0 (Control), BSN1 (5% DNP), BSN2 (10% DNP), BSN3 (15% DNP), BSN4 (20% DNP).

Polyphenols are well-recognized bioactive compounds with strong antioxidant activity, mainly represented by flavonoids and tannins^40^. Results in Fig. 1 (a and b) clearly showed that increasing levels of DNP in waffles significantly enhanced total phenolic (from 7.82 to 9.96 mg GAE/g) and flavonoid contents (from 0.83 to 1.34 mg RE/g), which was reflected in the improved DPPH scavenging activity, particularly in WN4. This increase can be attributed to the naturally high concentrations of phenolic acids, flavonoids, and tannins in Nabaq fruit^27^. A similar trend was observed in breadsticks, where TP, TF, and antioxidant activity gradually increased with rising DNP substitution, with BSN4 showing the highest values.

These findings are consistent with those of Coşkun, et al.^41^, who demonstrated that the incorporation of polyphenol-rich plant ingredients significantly boosts the antioxidant potential and functional quality of food products. Similarly, the study of Mandache, et al^42^. on bakery products fortified with fruit pomace confirmed that such enrichment effectively increased total phenolic content and antioxidant activity, which agrees with the trends observed in our work. Furthermore, Tolve, et al^43^. reported that fortifying wheat bread with grape pomace powder improved its nutritional, antioxidant, and sensory properties, in line with the improvements noted in our DNP-enriched waffles and breadsticks. In general, these comparisons reinforce the conclusion that DNP is a valuable functional ingredient capable of enhancing the bioactive profile and antioxidant potential of bakery products, aligning with the global shift toward healthier and functional food development.

### Color measurement of waffles, and breadsticks

**Fig. 2:**
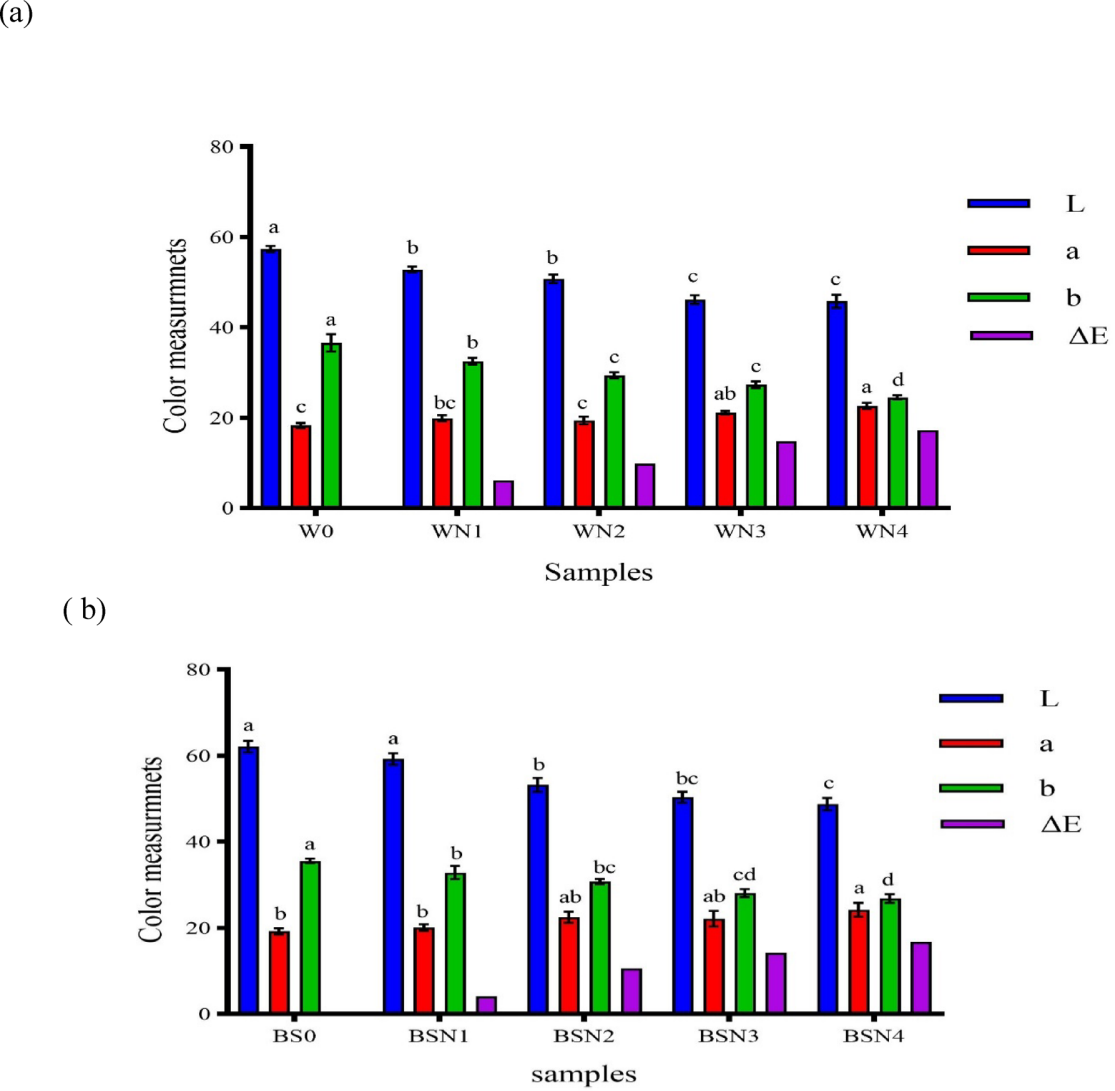
Color measurements of waffles **(a)**, Values represent the means ± standard deviation (*n* = 3). DNP = Dehydrated Nabaq powder, W0 (Control), WN1 (5% DNP), WN2 (10% DMP), WN3 (15% DNP), WN4 (20%DNP), Color measurements of breadsticks **(b)**, BS0 (Control), BSN1 (5% DNP), BSN2 (10% DNP), BSN3 (15% DNP), BSN4 (20% DNP).

Color is a significant sensory attribute affecting individuals’ inclination, acceptance, and purchasing decisions regarding food products. Waffles and breadsticks were analyzed for color in terms of lightness, redness, and yellowness values (Fig. 2). Among the waffle samples, the control (W0) recorded the highest lightness (57.36), followed by WN1 (52.85), with the lowest in WN4 (45.79). Meanwhile, WN4 exhibited the highest redness (22.63), surpassing the control’s 18.28. The control sample (W0) also displayed the highest yellowness (36.60) compared to those containing DNP (WN1–WN4).

Figure 2(b) shows that increasing DNP levels in breadsticks resulted in a decline in lightness values, from 62.18 in the control (BS0) to 48.74 in BSN4. Redness rose slightly, peaking at 24.23 in BSN4, while the control had the lowest value (19.21). Yellowness similarly decreased with increased DNP substitution.

Overall, increasing the level of DNP substitution in both waffles and breadsticks reduced lightness, leading to a darker color. This effect can be attributed to the natural pigments and phenolic compounds in Nabaq fruit, as well as an intensified Maillard reaction during baking, where reducing sugars interact with amino acids to produce browning compounds. The observed increase in redness may also be explained by the presence of red-brown pigments in Nabaq, including anthocyanins and other phenolics. Moreover, the ΔE values showed a gradual increase with higher substitution levels of dehydrated Nabaq powder in both waffles and breadsticks. This indicates a more pronounced overall color change compared to the control samples. The increase in ΔE can be attributed to the presence of natural pigments and phenolic compounds in Nabaq, as well as enhanced Maillard reactions during baking, which together contributed to the visible darkening of the products^30^. These results are consistent with the findings of These findings are in agreement with those reported by Altamim^30^, who observed that the control protein bar exhibited the highest lightness (L* = 65.30). Substituting glucose syrup with Egyptian Sidr (ES) pulp powder at levels of 5–15% significantly (*p* < 0.05) reduced L* values, thereby darkening the product. In terms of redness (a*), ES-fortified bars consistently showed higher values than the control (3.94), a change attributed to the presence of red-brown pigments and phenolic compounds. Likewise, yellowness (b*) values increased significantly with higher ES incorporation. Overall, these results confirm that the use of fruit-based powders can markedly modify the color characteristics of protein bars, with ES contributing to more intense red–yellow hues. Likewise, Srinivasan and Rana^44^found that anthocyanin-rich fruit-derived powders showed distinct color shifts under thermal treatments, especially increased redness and reduced lightness, depending on substitution levels. Lakshmikanthan, et al^45^. in their review emphasized similar observations across various fruit-rich food formulations, where increased pigment content led to visible darkening and hue changes. Tkaczyńska, et al^46^. studied anthocyanin stability under heating and noted that lightness decreased and redness increased in processed products with fruit powder. Such trends align strongly with our findings for waffles and breadsticks enriched with DNP, confirming that fruit pulp powders not only contribute to nutritional enhancement but also significantly alter color attributes in ways consistent with current literature.

#### Texture profile analysis of waffles, and breadsticks

**Fig. 3:**
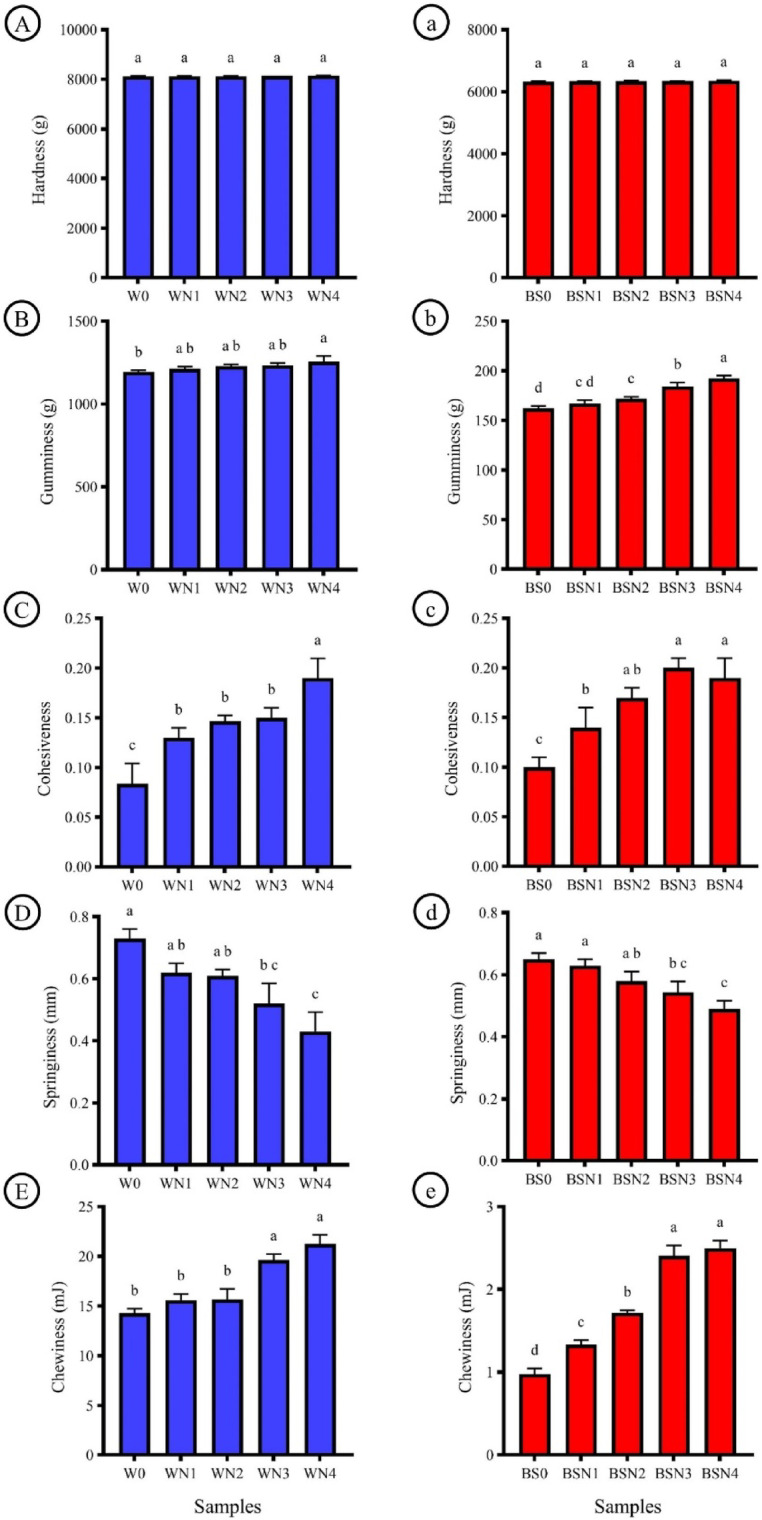
Texture profile analysis of waffles **(A-E)**, Values represent the means ± standard deviation (*n* = 3). DNP(Dehydrated Nabaq powder), W0 (Control), WN1 (5% DNP), WN2 (10% DMP), WN3 (15% DNP), WN4 (20%DNP), Texture profile analysis of breadsticks **(a-e)**, BS0 (Control), BSN1 (5% DNP), BSN2 (10% DNP), BSN3 (15% DNP), BSN4 (20% DNP).

Texture is a key determinant of food quality, strongly influencing consumer perception and acceptability. As shown in Fig. 3, the texture parameters of waffles and breadsticks including hardness, chewiness, gumminess, cohesiveness, and springiness were significantly (*p* < 0.05) affected by the incorporation of DNP. Increasing DNP levels resulted in notable rises in hardness, gumminess, cohesiveness, and chewiness, while springiness declined with higher substitution. The control waffle (W0) exhibited the lowest values for hardness, gumminess, cohesiveness, and chewiness. These changes are largely attributed to the high fiber and mucilage content of Nabaq fruit, which interferes with gluten network formation essential for elasticity and structure in wheat-based systems. The observed reduction in protein content may also weaken gluten starch interactions, further reducing elasticity. Additionally, the mucilage in Nabaq powder possesses binding properties that enhance cohesiveness and gumminess.

A similar pattern was observed in breadsticks, where increasing DNP levels significantly (*p* < 0.05) elevated hardness, gumminess, cohesiveness, and chewiness, while reducing springiness. The BSN4 sample recorded the highest values of these parameters compared to the control (BS0). these results differ from those reported by Altamim^30^, who reported that incorporating Egyptian Sidr (Nabq) pulp powder into protein bars significantly reduced hardness and cut resistance compared to the control. This reduction was attributed to the high fiber content of Nabq, which disrupts the protein matrix, increases moisture retention, and softens the overall texture. Such mechanisms align well with the present study, where higher levels of DNP in waffles and breadsticks increased gumminess and cohesiveness but reduced springiness, indicating that the fiber and mucilage in Nabq fruit play a key role in modifying gluten structure and overall textural quality. These findings are also in agreement with earlier reports (Altamim^30^,Coşkun, et al^41^.), which demonstrated that fruit- and fiber-based powders modify gluten development and substantially alter texture, often increasing hardness, gumminess, and cohesiveness due to their high fiber and polyphenol content.

#### Sensory evaluation of waffles, and breadsticks

Table (4) presents the sensory evaluation results of waffles. No significant differences (*p* > 0.05) were found in odor and taste between the control (W0) and waffles containing different levels of DNP. This stability can be attributed to the natural flavor-enhancing properties of Nabaq fruit, which is rich in sugars, organic acids, and phenolic compounds. However, at higher substitution levels, texture, overall acceptability, and color declined significantly (*p* < 0.05).

A similar trend was observed in breadsticks (Table 4), where increasing DNP levels caused significant (*p* < 0.05) decreases in taste, odor, texture, and overall acceptability compared with the control. These findings align with Hashem and Abd El-Lahot^27^reported that the sensory qualities of different products were affected by the substitution level of dehydrated Nabaq powder. Despite these declines, panelists still rated products containing Nabaq favorably, with average scores above 7 (“like very much”) at 50% substitution, while moderate acceptance (“like moderately”) was noted at higher or lower levels. The significant changes in color were mainly attributed to substitution levels and processing conditions during preparation.

In contrast, Altamim^30^reported that substituting glucose syrup with Egyptian Sidr (ES) or Saudi Sidr (SS) powders in protein bars enhanced sensory attributes compared with the control. At 10% replacement, texture achieved the highest scores, while taste and overall acceptability peaked at 15% ES or SS, with no significant differences between the two. These variations from the present study may reflect product-specific factors, as bakery systems (waffles and breadsticks) are more sensitive to fiber-induced changes in structure and color, whereas protein bar formulations benefited from the distinctive flavor and moisture retention properties of Sidr powders.

**Table 4:** Sensory evaluation results of waffles and breadsticks. DNP (Dehydrated Nabaq powder), W0 (Control), WN1 (5% DNP), WN2 (10% DNP), WN3 (15% DNP), WN4 (20% DNP), BS0 (Control), BSN1 (5% DNP), BSN2 (10% DNP), BSN3 (15% DNP), BSN4 (20% DNP). Values represent the means ± SD (on dry weight basis) of three independent replicates. Means within the same column followed by different letters are significantly different (*p* < 0.05). All sensory scores were recorded within the 1–9 scale range; minor exceedances in mean ± SD are due to rounding effects during data processing.

Product	Odor	Taste	Color	Texture	Overall acceptability
Waffles					
W0 WN1 WN2 WN3 WN4	8.9 ± 0.32 ^a^ 8.9 ± 0.32 ^a^ 8.8 ± 0.42 ^a^ 8.8 ± 0.46 ^a^ 8.6 ± 0.70 ^a^	9.0 ± 0.00 ^a^ 8.9 ± 0.32 ^a^ 8.7 ± 0.48 ^a^ 8.6 ± 0.52 ^a^ 8.6 ± 0.52 ^a^	9.0 ± 0.00 ^a^ 8.8 ± 0.42 ^ab^ 8.5 ± 0.53 ^b^ 8.1 ± 0.57 ^c^ 7.9 ± 0.32 ^c^	9.0 ± 0.00 ^a^ 8.9 ± 0.32 ^a^ 8.5 ± 0.46 ^b^ 8.4 ± 0.52 ^b^ 7.9 ± 0.57 ^c^	9.0 ± 0.00 ^a^ 8.9 ± 0.32 ^a^ 8.6 ± 0.52 ^b^ 8.1 ± 0.57 ^b^ 8.0 ± 0.30 ^c^
Breadsticks					
BS0 BSN1 BSN2 BSN3 BSN4	8.9 ± 0.32 ^a^ 8.8 ± 0.42 ^a^ 8.8 ± 0.42 ^a^ 8.6 ± 0.52 ^ab^ 8.2 ± 0.92 ^b^	9.0 ± 0.00 ^a^ 8.9 ± 0.32 ^ab^ 8.5 ± 0.53 ^b^ 8.5 ± 0.53 ^b^ 8.5 ± 0.53 ^b^	9.0 ± 0.00 ^a^ 8.8 ± 0.42 ^ab^ 8.4 ± 0.70 ^bc^ 8.0 ± 0.67 ^cd^ 7.7 ± 0.48 ^d^	9.0 ± 0.00 ^a^ 8.8 ± 0.42 ^ab^ 8.4 ± 0.69 ^bc^ 8.3 ± 0.48 ^c^ 7.7 ± 0.67 ^c^	9.0 ± 0.00 ^a^ 8.8 ± 0.53 ^ab^ 8.5 ± 0.71 ^b^ 8.0 ± 0.67 ^c^ 7.9 ± 0.32 ^c^

### Constraints and prospective directions

This study provides valuable insights into the use of *Ziziphus spina-christi* (Sidr) fruit powder as a functional ingredient in waffles and breadsticks. Nevertheless, some limitations should be acknowledged. The current analyses primarily focused on the nutritional composition, total phenolic and flavonoid contents, antioxidant activity, texture, color, and sensory characteristics. However, advanced characterization techniques such as HPLC for compound profiling, in vitro digestion for evaluating bioaccessibility, and microstructural analysis were not included.

In addition, while the mineral content was quantified, the study did not assess the presence of anti-nutritional factors such as phytates, oxalates, saponins, and tannins, which can significantly influence the bioavailability of minerals. Moreover, the sensory evaluation was conducted on a relatively small consumer panel, which may not fully represent broader consumer preferences.

Future research should expand the analytical scope to include HPLC-based phytochemical profiling, in vitro digestion and bioavailability studies, as well as in vivo or clinical trials to confirm potential health benefits such as anti-diabetic and gut-health-promoting effects. Evaluating the stability of bioactive compounds and antioxidant activity during storage—along with monitoring peroxide value and physicochemical changes—will be essential to determine the shelf-life and oxidative stability of Sidr-fortified bakery products. Furthermore, future studies should examine compositional variations in Sidr fruit due to regional, seasonal, and processing differences and explore combinations with other functional ingredients (e.g., legumes, seeds, or dietary fibers) to further enhance the nutritional profile and consumer acceptability of functional bakery products.

## Conclusion

This study demonstrates the potential of dehydrated *Ziziphus spina-christi* fruit powder (DNP) as a functional ingredient in bakery products such as waffles and breadsticks. The powder itself contained 16.23% moisture, 5.86% protein, 1.06% fat, 5.54% ash, 4.21% fiber, 83.33% carbohydrates, and provided 366.58 kcal/100 g. Incorporation of DNP significantly enhanced ash, crude fiber, phenolics, flavonoids, and antioxidant capacity, while higher substitution levels reduced protein content, lightness, and some sensory attributes due to its fiber, mucilage, and pigment content. Sensory evaluation confirmed that moderate incorporation levels (10–15%) achieved the best balance between nutritional enhancement and consumer acceptability. Beyond the tested bakery products, DNP shows promise for broader applications in functional foods, including snacks, cereals, and dairy-based formulations. Future research should focus on shelf-life evaluation, bioavailability, and clinical validation to support the wider adoption of DNP in functional food development.

## Data Availability

All data generated or analysed during this study are included in this published article.
